# Same Chance of Accessing Resection? Impact of Socioeconomic Status on Resection Rates Among Patients with Pancreatic Adenocarcinoma—A Systematic Review

**DOI:** 10.1089/heq.2019.0099

**Published:** 2021-03-22

**Authors:** Alexandre Thobie, Andrea Mulliri, Véronique Bouvier, Guy Launoy, Arnaud Alves, Olivier Dejardin

**Affiliations:** ^1^ Department of Digestive Surgery, University Hospital of Caen, Caen Cedex, France.; ^2^ UMR INSERM 1086 UCN ‘ANTICIPE,’ Caen, France.; ^3^ Registre des Tumeurs Digestives du Calvados, Caen, France.; ^4^ Department of Research, University Hospital of Caen, Caen Cedex, France.

**Keywords:** pancreatic adenocarcinoma, socioeconomic status, surgery

## Abstract

**Background:** The incidence of pancreatic cancer is growing and the survival rate remains one of the worst in oncology. Surgical resection is currently a crucial curative option for pancreatic adenocarcinoma (PA). Socioeconomic factors could influence access to surgery. This article reviews the literature on the impact of socioeconomic status (SES) on access to curative surgery among patients with PA.

**Methods:** The EMBASE, MEDLINE, Web of Science, and Scopus databases were searched by three investigators to generate 16 studies for review.

**Results:** Patients with the lowest SES are less likely to undergo surgery than high SES. Low income, low levels of education, not being insured, and living in deprived and rural areas have all been associated with decreased rates of surgical resection. Given the type of health care system and geographic disparities, results in North American populations are difficult to transpose to European countries. However, a similar trend is observed in difficulty for the poorest patients in accessing resection. Low SES seems to be less likely to be offered surgery and more likely to refuse it.

**Conclusions:** Inequalities in insurance coverage and living in poor/lower educational level areas are all demonstrated factors of a lower likelihood of resection populations. It is important to assess the causal effect of socioeconomic deprivation to improve understanding of this disease and improve access to care.

## Introduction

Pancreatic adenocarcinoma (PA) is currently the twelfth most common cancer in the world and is one of the most lethal.^1^ It is forecast to become the second most important cause of cancer mortality by 2030.^2^ An increase in risk factors like smoking^3–5^ and chronic medical conditions such as diabetes mellitus,^4,6,7^ chronic pancreatitis,^7^ and obesity^8^ are associated with growing incidence. Since its mortality equals its incidence, a major question is to find a potential curative treatment for this cancer. Even though 5-year survival is dramatically poor for PA with fewer than 5% of survivors, survival among resected patients has been reported to be 20%.

For nontreated patients with PA, the key to curative therapy is resection, yet only 15–20% of patients with PA are resectable. A major challenge is to increase the number of resectable patients to improve access to curative treatment, yet diagnosis is often made late with 50% of PA diagnosed at a metastatic stage.

The question of the influence of social environment in PA is not widely accepted, with a social gradient of higher mortality and higher morbidity from highest to lowest affluence level. This effect is present in both low-income countries as well as high-income ones. Social inequalities could affect the management of each patient at different steps. At patient level, awareness of symptoms, stage, and living and working conditions are potential sources of inequalities in care. The health care system may also be responsible for inequalities when deprived patients are either not referred to the same center, are subjected to a long delay between presentation and diagnosis, or do not benefit from the same management guidelines. Although these aspects were regularly investigated for almost every cancer localization and regularly highlighted as an important determinant of cancer outcomes, such influences of cancer inequalities were not deeply investigated for PA, potentially due to the dramatically poor survival for PA. Nonetheless, when resected, the survival of PA cancer is comparable to other localizations in which social inequalities play role, such as lung, liver, biliary tract, and esophagus.

Health systems are notably different between Europe and the United States, so they have a different impact on access to health care and its social determination. In the United States, health disparities in cancer have been found to be linked with insurance coverage.^9,10^ The sociodemographic environment has an influence relatively early on the cancer care continuum, affecting presentation and therefore influencing which patients undergo surgical resection. Several studies have shown that non-medical factors such as insurance status,^11^ socioeconomic status (SES), place of residence, and provider characteristics^11^ are associated with disparities in treatment and survival rates of PA.^12–14^ This systematic review analyzed the impact of SES on access to curative surgery among patients with PA.

## Methods

### Selection of studies

Articles included in the review were selected using MEDLINE, Web of Science, and Scopus databases with the following MESH terms: pancreatic neoplasm, SES, social classes, curative, and resection. Selection was restricted to English-language articles indexed from database inception to October 15, 2018. The search retrieved 114 abstracts that were carefully reviewed by two oncologic surgeons and one epidemiologist for clinical relevance. Two narrative reviews were excluded. The bibliographies of all full-text articles selected were manually searched to identify any additional study that might be relevant. To be included in the final selection, articles had to be conducted on PA, as defined by the International Statistical Classification of Diseases for Oncology, describing access to oncological resection with curative intent. Articles with results on surgery without any information on curative intent were excluded.

### Definition of SES

The definition of SES and its mode of assessment, when specified, varied greatly between articles. Some articles relied on unique parameters such as income, occupation, or insurance status to categorize their population. Others used a score combining several parameters, which is more relevant to assess the complexity of SES, which is classically defined by cultural, financial, and social dimensions. U.S. studies often incorporate an ethnic dimension because of disparities between racial groups. Since this factor was not comparable with European studies, studies that used ethnic dimension as socioeconomic parameter were excluded. Moreover, a growing number of articles used an aggregated approach to integrate items pertaining to geographical context. We therefore considered articles that used any of the following as proxies for socioeconomic factors: income, insurance status, level of education, area of residency, and deprivation index (when explained).

**FIG. 1.**
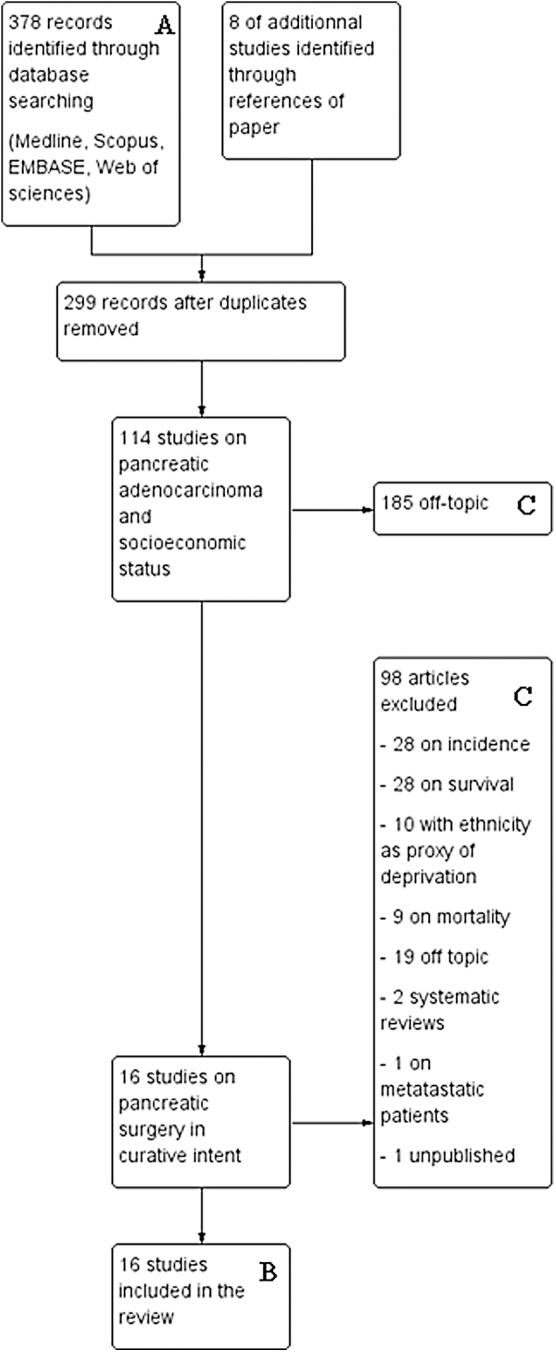
Flowchart describing the study selection process. The flowchart explains the study selection process from initial research in databases **(A)** to final inclusion **(B)**. *Boxes* **(C)** on the *right*, throughout the process, explain the reasons for exclusion of studies.

Finally, 16 suitable studies were identified for review: 10 based on populations from the United States, 1 from United Kingdom, 1 from Canada, 1 from France, and 2 from the Netherlands (Fig. 1 shows the process).

The submission has received all authors' Internal Review Board approvals.

## Results

Table 1 summarizes studies on the links between SES and access to surgery. Most concern U.S. cancer registries. The results concerning the impact of deprivation on resection rates are globally similar: socioeconomic deprivation is associated with a lower likelihood of access to resection. At individual and institutional level, various factors explain these inequalities.

**Table 1.** Studies Describing Impact of Socioeconomic Status on Access to Resection Among Patients with Pancreatic Adenocarcinoma

Study	Country	Period of study	Population (n)	Type of variables for SES	Calculation of SES	Access to resection
Shavers^16^	United States	1998	2404	Individuals	Insurance status: no insurance, private insurance only, HMO or other managed care plan, Medicaid/Medicare only and other insurance	Uninsured less often were recommended for or received surgery (OR 0.09 [0.01–0.62]) and (OR 0.07 [0.01–0.49]).
Seyedin^28^	United States	1988–2002	5908	Individuals	Median family income	Low and middle income vs. high income: ORa 0.52 [0.35–0.78] and ORa 0.83 [0.71–0.97] respectively.
Shah^27^	United States	1988–2009	35,944	Individuals	Family below poverty, Education below High school, rural/urban residency	Areas with lower educational attainment: adjusted OR 1.011 [1.005–1.018], *p*=0.001 Areas of higher poverty: adjusted OR 0.969 [0.959–0.979], *p*<0.001.
Abraham^17^	United States	1994–2008	20,312	Individuals	Insurance Status: Medicaid, Non Medicaid/Medicare, Medicare, None, Unknown.	Non-Medicare/Medicaid vs. Medicaid OR 1.7 [1.4–2.2] Medicare vs. Medicaid OR 1.8 [1.4–2.4].
Shapiro^11^	United States	2004–2011	17,530	Individuals	Insured/Noninsured status, marital status	Insured vs. uninsured: OR 1.63 [1.22–2.18]
Markossian^22^	United States	2013	245	Individuals	Area of residency: small rural or isolated, large rural, urban	No differences between areas of residency (*p*=0.115).
Chang^18^	United States	2006–2014	2103	Individuals	Income, High school graduate, insurance status	Medicare more likely to undergo surgery: adjusted OR 1.72 (1.14–2.59) *p*=0.01.
Cress^19^	United States	1994–2000	10,612	Collective	Block-group quintiles based on statewide measurement of SES (Census data by block-group of residence)	HSES vs. LSES (48% versus 44%, *p*=0.02).
Bilimoria^15^	United States	1995–2004	9559	Collective	Median household income and education assessed at the zip code level based on U.S. Census data insurance (individuals)	Lower annual incomes vs. higher (OR 0.56 [0.33–0.94]), less education, were on Medicare or Medicaid (OR 0.63 and 0.78) (*p*<0.0001). More likely to refuse if on Medicaid insurance (*p*<0.0001).
Zell^20^	United States	1989–2003	17,326	Collective	5 quintiles: single index variable in California Cancer Registry using statewide measures of education, income, and occupation from census data	LSES vs. HSES: 9.9% vs. 12.4%, *p*=0.02.
Cheung^12^	United States	1998–2002	2877	Collective	Divided in 4 quartiles of area of poverty	LSES vs. HSES: 16.5% vs. 19.8% *p*<0.001.
Kagedan^21^	Canada	2005–2010	6296	Collective	Ontario Marginalization Index (ON-Marg): 4 dimensions: residential instability, material deprivation, dependence and ethnic concentration. Each area of marginalization was divided into quintiles	Rural areas (OR 0.68 [0.51–0.91]) and urban areas with lower incomes (OR range [0.49–0.77]) vs. urban areas with higher incomes. Material deprivation (OR 0.86 [0.79–0.94]), increasing levels of residential instability (OR 0.86 [0.80–0.94])
Coupland^25^	UK	2005–2009	31,973	Collective	Income domain of indices of deprivation, Divided in 5 quintiles	Most affluent vs. most deprived: 9.1% vs. 7.2% (*p*<0.001).
Van Roest^24^	Netherlands	1989–2011	34,757	Collective	SES score provided by the Netherlands Institute for Social Research, divided in three groups	HSES vs. LSES: 10% vs. 9% (*p*=0.006).
Bakens^23^	Netherlands	2005–2013	698	Collective	Neighborhood level using postal codes combined with mean value of housing and mean household income (Netherlands Statistics Agency) divided into 3 categories.	Low and intermediate SES (32% vs. 37%) vs. HSES (48%) (*p*=0.002; multivariable analysis: LSES: OR 0.63 [0.40–0.98]; intermediate SES: OR 0.62 [0.42–0.92]).
Thobie^26^	France	2000–2014	1451	Collective	European Deprivation Index, divided in 5 quintiles	Less deprived areas vs. more deprived areas: ORa 1.73 [1.08–2.47], *p*=0.013.

HMO, Health Maintenance Organization; HSES, high socioeconomic status; LSES, low socioeconomic status; OR, odds ratio; ORa, adjusted odds ratio; SES, socioeconomic status.

### Status of insurance

Insurance status is a predictor of undergoing resection in the United States: uninsured patients and those on non-Medicare/Medicaid are less likely to receive curative surgery than patients on private insurance and Medicaid.^11,15–17^ Abraham et al. using the California Cancer Registry found that patients on non-Medicare/Medicaid, Medicare and of unknown insurance status were more likely to undergo surgery than those on Medicaid, after adjustment for patients characteristics and tumor factors.^17^ A similar effect was found for both non-Medicare/Medicaid and Medicare patients. Only the lowest SES, that is, those with Medicaid and having no insurance, had a lower access to resection. This finding was confirmed by Shapiro et al.^11^ Using the surveillance, epidemiology, and end results (SEER) registry, they found that being insured, irrespective of the type of insurance program, was an independent predictor of being more likely to be resected in multivariate analysis among resectable patients. Only Bilimoria et al. using the National Cancer Data Base found no difference in resection rates between privately insured and uninsured persons, but only 1.8% in that study were not insured.^15^ Another issue is refusal of surgery, beneficiaries of Medicaid and Medicare refusing it more than persons with private insurance. Patients receiving Medicare might have been more reticent to be operated because most of them were older than 65 years. On the other hand, Chang et al. found that beneficiaries of Medicare were more likely to receive surgery than persons not on Medicare.^18^

### Area of poverty

In summary, insurance status is an established predictor of access to resection in U.S. studies. Persons with the lowest SES, as identified by noninsured and Medicare/Medicaid status, are less likely to undergo surgery than non-Medicare/Medicaid status.

Living in a resource poor area also seems to be associated with less access to resection in the United States.^12^ Four studies conducted on U.S. registries used place of residency with the zip code to score SES.^12,15,19,20^ Results from these four registry studies were relatively homogeneous since patients living in the areas with the high deprivation index scores were less operated than those living in the lower deprivation index scores. An interesting finding was the proportional/dose effect of median income areas in the study by Bilimoria et al.: the poorer the area of residency, the lesser the access to resection (*p*<0.0001) and the greater the risk of refusing surgery (*p*=0.04). A similar significant trend was found in the same study in persons with a lower level of education (*p*<0.0001). More people had no insurance or benefited from a health program for the poorest like Medicaid/Medicare in the poorest areas.^12^ Only Cheung et al. found a difference in tumor characteristics: more patients in poorer areas had tumors >5 cm, but no differences were observed with regard to stage, differentiation, and lymph node status.^12^ However, in that study, persons with low SES were older and more of them were smokers, two factors that impact access to surgery.

### Geographic disparities

An important factor not taken into account by the notion of areas of poverty is geographic remoteness. Although they did not take the distance to the specialized center into account, Kagedan et al. divided their population into rural and urban areas.^21^ In urban areas, persons in the highest urban income quintile were more likely to be resected than those in the other quintiles. After adjustment on age comorbidity and geographic region, patients living in rural areas also had a significantly lower likelihood of undergoing surgical resection than those living in the highest urban income neighborhoods.^21^ Increasing levels of residential instability (odds ratio, OR 0.86 [0.80–0.94]) and material deprivation (OR 0.86 [0.79–0.94]) predicted a decreased likelihood of undergoing resection, but the two other dimensions did not. In another study, Markossian et al. found no difference in univariate analysis between rural and urban areas with regard to receiving surgery.^22^

The issue of socioeconomic deprivation has received little attention in Europe. Among metastasis-free patients, it was found that low SES (low median income based on zip code) patients were more likely to undergo laparotomy (probability of resection was not analyzed) than high SES ones in multivariate analysis: adjusted OR 0.63 [0.40–0.98].^23^ High SES patients had more advanced stages at diagnosis as suggested by results for stage: 80% of stage II/III among high SES versus 65% among low SES (*p*=0.002). Tumor stage was not a predictor of resection in multivariable analysis probably because there were nonresectable patients among those with stages II/III. Patients with a high SES were diagnosed more in specialized pancreatic centers (*p*=0.026). Others studies in Dutch registry and English National Cancer Data Repository found slightly better access to resection for patients from high SES neighborhoods (10% vs. 9%, *p*=0.006 and 9.1% vs. 7.2%, *p*<0.001, respectively).^24,25^ On a specialized registry, Thobie et al. showed that living in less deprived areas was a predictor of resection in multivariable analysis (ORa 1.73 [1.08–2.47], *p*=0.013).^26^

A study on the SEER registry by Seyedin et al., concerning resectable cases only, is in line with the previous study. Patients with low or middle incomes were less likely to undergo resection than those with a high income: ORa 0.52 [0.35–0.78] and ORa 0.83 [0.71–0.97], respectively. There were significantly more regional diseases (stages IIA-IIB) among low (51.2%) and middle SES (50.4%) patients than among those with high SES (43.3%) and less localized disease (*p*=0.001).

### Therapeutic decisions

Three hypotheses to explain nonaccess to surgery have been put forward by Shah et al.: not being resectable, refusing surgery if recommended, and not undergoing surgery if recommended for.^27^ Patients from areas with a lower educational level were more likely to undergo resection (adjusted OR 1.011 [1.005–1.018], *p*=0.001), more likely to be resected if given the offer (adjusted OR 1.037 [1.026–1.049], *p*<0.001), and more likely to refuse surgery (adjusted OR 1.03 [1.01–1.05], *p*=0.005). Patients living in areas of greater poverty were less likely to undergo resection (adjusted OR 0.969 [0.959–0.979], *p*<0.001) and less likely to be resected if given the offer (adjusted OR 0.960 [0.945–0.976], *p*<0.001), but were not more likely to refuse it than those living in areas, where there was less poverty. These findings suggest that the poorest patients are less resected, even if it is recommended for them and they are offered it. However, they do not seem to refuse surgery more than persons with the highest SES. Patients from areas with a lower educational level are slightly more likely to be resected and more likely to refuse.

Inequalities in insurance coverage, living in remote and poor areas, and having a lower educational level are all demonstrated factors of a lower likelihood of resection in American and Canadian populations. Because the health care systems are different and given the urban and geographic disparities, results from North American populations are difficult to transpose to European countries. Despite these differences a similar trend is observed in difficulty of accessing resection in the poorest patients.

## Discussion

Inequalities in access to care in pancreatic cancer are a global concern, but this subject has received poor consideration in Europe. Studies have been essentially led on U.S. registries. Low income,^15,21,28^ low levels of education,^15^ not being insured,^11,15–17,29^ and living in deprived and rural areas^21,24–27^ have all been associated with decreased rates of surgical resection. People from low socioeconomic groups are therefore less likely to receive surgery than those from higher classes. Several explanations can be advanced: factors related to patient and disease, and factors related to health care system and providers.

### Factors related to patients and disease

#### Resectability

Results are discording. Most of the studies have shown no clear difference on stage at presentation or resectability status between low and high SES.^11,26,30,31^ Patients who are non-Medicare/Medicaid are slightly more likely to have a resectable disease.^17^ Others studies found that patients in the lowest socioeconomic groups are more likely to present with a tumor >5 cm or with more advanced diseases.^12,28^ On the contrary, it has been found that patients with low SES had slightly more local disease, less disease beyond the pancreas, and a lower stage than those with high SES.^23,24,26^ Resectability for PA is different from TNM staging as defined by UJCC classification. Resectability has been defined for tumors with no arterial tumor contact (celiac axis, superior mesenteric artery, or common hepatic artery), no tumor contact with the superior mesenteric vein, or portal vein or <180° contact without vein contour irregularity.^32^ Heterogeneity in these results could be explained by heterogeneity in staging and grading PA among the studies. Resectability should be the main criterion for staging PA because it determines access to resection and through curative intent treatment.

#### Operability: comorbidities and decisions

Even with equal resectability status, the opportunities are not the same for everyone. Low-income patients, rural inhabitants, and uninsured/Medicaid patients are not offered surgery as often as the richest patients, even when their tumor is considered resectable.^15,28^ This may be because they have more contraindications to surgery because of comorbidities and chronic diseases.^33,34^ Those from lower socioeconomic groups are less likely to follow recommendations for treatment.^35^ On the California Cancer Registry, it has been shown that in lower SES patients, the less NCCN guidelines were followed.^35^ These disparities were directly associated with poorer survival in patients for whom recommendations were not followed. On the other hand, low SES patients are less resected, even if it is recommended and they are offered it, but they do not refuse surgery more than the highest SES. Patients from areas with a lower educational level are more likely to refuse.^27^ Lack of education, understanding, and information may also lead to unwillingness to undergo surgery.

### Factors related to health care system and providers

#### Delays of access to treatment

PA evolves rapidly so time is of essence. In a study assessing time between first symptoms and first consultation, SES was not associated with longer delays until consultation.^36^ Patients with the lowest SES had a longer treatment time between consultation and treatment in univariate analysis, almost reaching significance in multivariable analysis.

#### Access to specialized centers

Access to specialized centers for diagnosis seems to be impacted by SES in Europe. Patients with high SES are significantly more diagnosed in specialized centers (≥20 pancreatoduodenectomies annually) than those with low SES.^23^ Patients were more likely to undergo surgery in such centers than those diagnosed elsewhere. Being diagnosed in a pancreatic center probably gives patients more likelihood of being cured. Each case is discussed in a multidisciplinary meeting with at least a hepatobiliary surgeon, a radiologist, and an oncologist, as recommended by ASCO and ESMO.^32,37^ Patients with high SES are more likely to be addressed to high-volume centers (HVC).^12^ Beyond the obvious geographical disparities linked to distance, socioeconomic disparities may impact access to specialized centers and to consultations with specialists. Patients with lower SES are less likely to be treated in high-volume centers and teaching facilities (TFs).^12^ Whatever the extension of the tumor, high SES patients had more access to HVC and TFs. Patients from rural areas are significantly less likely to see a medical oncologist than urban residents.^22^ Globally, the results suggested that SES impacted access to specialized centers and consultations with specialists. Only one study has examined the impact of SES on margin, reporting lower R0 resection rates among lower SES.^38^

Postoperative morbidity remains high in pancreatic surgery. Patients from low socioeconomic groups are more likely to have operations in an low volume hospital,^39^ which is an independent risk factor for poor outcomes in pancreatic surgery.^40,41^ Lower 30-day mortality rates among those from higher socioeconomic groups have been found.^12^ However, it is important to understand how and why SES could affect surgical morbidity. Access to adjuvant therapy may be affected by SES, because the poorest people are less likely to receive chemotherapy.^12,33,37,42^

#### Survival following resection

Lower overall survival rates following resection have been highlighted for those from lower socioeconomic groups.^12,19,38^ Lin et al. found a huge gap in 5-year survival rates.^38^ Two other studies,^12,19^ both conducted in the United States, found lower median overall survival for low SES.^12,19^ On the contrary, on a French registry, no impact of SES on survival following resection was found in multivariable analysis, suggesting that SES impacted essential access to resection.

The different ways of scoring SES and score create difficulties in comparisons. Using a validated index could reduce problems of comparability.^43,44^ The health system could play also a major role in this effect. In a system where payment for cancer-related care depends on insurance status, as in the United States, decisions about the therapeutic course could depend on SES. Donelan et al. noted that access to care is more difficult in the United States than in Germany, where there is a universal multipayer system.^45^ Too few studies have been carried out on the subject in Europe where the mechanisms of inequalities are not the same because of different health systems and different living standards.

One of the major challenges in the management of pancreatic cancer is access to resection. Since there is no screening test for specific symptoms associated with early-stage disease, inequalities seem to have little impact on presentation and timing of presentation. Rather, they affect access to resection through access to specialist consultations, specialists' and patients' decision, and the application of recommendations and access to specialized centers. These points need to be improved by setting up a detailed network for the management of PA, the systematic discussion of each case by a multidisciplinary college of specialists, and providing appropriate information to decrease refusal rates.^27^ Ensuring access to surgical resection is a key step toward improving equity in the treatment of PA, even within a universal health care system. SES also has an effect on both the likelihood of receiving adjuvant treatment and subsequent survival. The impact of SES on patients with pancreatic cancer is an important factor for increasing the understanding of this disease and improving care.

## Abbreviations Used

HMO: Health Maintenance Organization
HSES: high socioeconomic status
LSES: low socioeconomic status
OR: odds ratio
ORa: adjusted odds ratio
PA: pancreatic adenocarcinoma
SEER: surveillance, epidemiology, and end results
SES: socioeconomic status
TFs: teaching facilities
